# Transcriptional Differential Analysis of Nitazoxanide-Mediated Anticanine Parvovirus Effect in F81 Cells

**DOI:** 10.3390/v16020282

**Published:** 2024-02-12

**Authors:** Xia Su, Hongzhuan Zhou, Ziwei Han, Fuzhou Xu, Bing Xiao, Jin Zhang, Qi Qi, Lulu Lin, Huanhuan Zhang, Songping Li, Bing Yang

**Affiliations:** 1 Beijing Key Laboratory for Prevention and Control of Infectious Diseases in Livestock and Poultry, Institute of Animal Husbandry and Veterinary Medicine, Beijing Academy of Agriculture and Forestry Sciences, Beijing 100097, China; sxia2013@163.com (X.S.); hanfu2002@sohu.com (H.Z.); hzwyh513@163.com (Z.H.); fuzhouxu@163.com (F.X.); xiaobing1972@sohu.com (B.X.); 17710300295@163.com (J.Z.); q1258840198@163.com (Q.Q.); 18601147804@163.com (L.L.); 15845814273@163.com (H.Z.); 2 School of Life Science and Biopharmaceutics, Shenyang Pharmaceutical University, Shenyang 110016, China

**Keywords:** canine parvovirus, nitazoxanide, RNA-seq, cell cycle

## Abstract

Canine parvovirus (CPV) is a single-stranded DNA virus that can cause typical hemorrhagic enteritis, and it is one of the common canine lethal viruses. In previous studies, we screened the Food and Drug Administration (FDA)’s drug library and identified nitazoxanide (NTZ), which has anti-CPV capabilities. To investigate the potential antiviral mechanisms, we first reconfirmed the inhibitory effect of NTZ on the CPV by inoculating with different doses and treating for different lengths of time. Then, the differences in the transcription levels between the 0.1%-DMSO-treated virus group and the NTZ-treated virus group were detected using RNA-seq, and a total of 758 differential expression genes (DEGs) were finally identified. Further Gene Ontology (GO) and Kyoto Encyclopedia of Genes and Genomes (KEGG) enrichment analyses of the DEGs revealed that these genes are involved in a variety of biological processes and/or signaling pathways, such as cell cycle, mitosis and cell proliferation and differentiation. A protein–protein interaction (PPI) analysis further identified hub genes associated with cell cycle and division among the DEGs. In addition, the expression levels of some of the enriched genes were detected, which were consistent with the high-throughput sequencing results. Moreover, when the cell cycle was regulated with cell cycle checkpoint kinase 1 (Chk1) inhibitor MK-8776 or Prexasertib HCl, both inhibitors inhibited the CPV. In summary, the transcriptome differential analysis results presented in this paper lay the foundation for further research on the molecular mechanism and potential targets of NTZ anti-CPV.

## 1. Introduction

Among canine diseases, viral diarrhea has caused serious risk to the dog industry and dogs due to its complex etiology, high morbidity and mortality [1,2,3]. Canine parvovirus (CPV) is one of the most dangerous infectious agents of diarrhea in young dogs. The clinical characteristics of infected dogs are vomiting, fever, diarrhea, hemorrhagic enteritis, myocarditis and leukopenia [4,5,6]. In addition, CPV can also infect several wild carnivores and even noncarnivores [7,8]. CPV is a negative single-stranded DNA belonging to the family Parvoviridae, genus Protoparvovirus. The CPV genome is approximately 5 kb in length and contains two open reading frames (ORFs), which encode nonstructural proteins (NS1 and NS2) and structural proteins (VP1 and VP2) [9]. Canine parvovirus spreads widely and evolves rapidly. In clinic, it mainly depends on vaccination for immune prevention [4]. At present, there are no reports of antiviral small molecule drugs for the specific treatment of CPV.

An increasing number of studies have focused on alternative strategies for CPV treatment and have reported a number of potential active antivirals against CPV for evaluation, such as interferon-ω [10], lithium chloride [11], and phosphorylated radix cyathulae officinalis polysaccharide [12]. In a previous study, we found that nitazoxanide (NTZ) was able to inhibit CPV replication in vitro [13]. NTZ is a nitrothiazole benzamide derivative (2-acetyloxy-*N*-5-nitro-2-thiazolyl). In 2002, the US Food and Drug Administration (FDA) approved NTZ as an oral antiparasitic drug [14]. NTZ also has antibacterial and antiviral activities [15,16]. NTZ can inhibit not only RNA viruses such as influenza A virus [17], hepatitis C virus [18] and human immunodeficiency virus (HIV) [19] but also some DNA viruses, including human cytomegalovirus (HCMV) [20] and hepatitis B virus (HBV) [21]. Its antiviral mechanisms are diverse, while the molecular mechanism by which NTZ inhibits CPV remains unclear.

In this study, based on the confirmation that NTZ inhibits CPV in vitro, high-throughput transcriptome sequencing technology was used to analyze the differences in transcription levels between the 0.1%-DMSO-treated virus group and the NTZ-treated virus group. The differential genes were further analyzed by GO, KEGG and PPIs. This research lays the foundation for the study of the molecular mechanism and potential targets of NTZ anti-CPV.

## 2. Materials and Methods

### 2.1. Cell and Virus

The feline kidney fibroblast-like monolayer cell line (F81) was purchased from the American Type Culture Collection (ATCC). F81 cells were grown in DMEM (Gibco, Grand Island, NY, USA) supplemented with 10% fetal bovine serum (Gibco, Grand Island, NY, USA), 100 U/mL penicillin and 0.1 mg/mL streptomycin and cultured at 37 °C and 5% CO_2_. The New CPV-2a strain SD6 (297Ala, 426Asn) was isolated and identified by our laboratory, and its VP2 coding gene was registered in GenBank as MN101724.

### 2.2. Reagents and Antibodies

Nitazoxanide (NTZ) and the cell cycle checkpoint kinase 1 (Chk1) inhibitors MK-8776 and Prexasertib HCl were purchased from Selleck Chemicals (Selleck, Houston, TX, USA). The anti-VP2 monoclonal antibody was purchased from Ingenasa (Ingenasa, Madrid, Spain). Beta-actin monoclonal antibody was purchased from Thermo Fisher (Thermo Fisher, Waltham, MA, USA). The ProteinExt^®^ Mammalian Total Protein Extraction Kit was purchased from TransGen (TransGen Biotech, Beijing, China). Total RNA extraction was conducted using the RNeasy Mini Kit (QIAGEN, Hilden, Germany). The reverse transcription kit FastKing RT Kit (With gDNase) and quantitative PCR kit SuperReal PreMix Plus (SYBR Green) were purchased from TIANGEN (TIANGEN, Beijing, China).

### 2.3. Validation of Anti-CPV Effect of NTZ In Vitro

F81 cells were inoculated into a 6-well plate with 7.5 × 10^5^ cells per well, and cells were pretreated with a final concentration of 10 µM NTZ (dilute 10 mM NTZ stock solution dissolved in DMSO 1000 times) for 1 h, while control cells were pretreated with 0.1% DMSO (dilute DMSO solution without NTZ 1000 times). Cells were inoculated with different doses of New CPV-2a strain SD6 (297Ala, 426Asn) at 0.1, 0.5 and 2.5 MOI, and cell samples were collected 48 h later. Alternatively, cells were inoculated with 0.1 MOI of New CPV-2a strain SD6 (297Ala, 426Asn) after NTZ pretreatment of the cells for 1 h, and cell samples were collected at 24 h, 48 h and 72 h postinoculation.

### 2.4. Western Blot

Western blot was used to detect the cell samples inoculated with different doses of the SD6 strain, as well as the cell samples collected at 24 h, 48 h and 72 h postinoculation (with an inoculation dose of 0.1 MOI). By detecting the expression level of the VP2 protein, the inhibitory effect of NTZ on different doses of CPV and different times after CPV inoculation was verified. Briefly, the collected cells were lysed with ProteinExt^®^ Mammalian Total Protein Extraction Kit (TransGen Biotech, Beijing, China), lysed on ice for 20 min and centrifuged at 14,000 rpm for 20 min at 4 °C. The protein concentration of the supernatant was determined with the Pierce™ BCA Protein Assay Kit (Thermo Fisher, Waltham, MA, USA). The equivalent amount of cell lysate was analyzed using sodium dodecyl sulphate polyacrylamide gel electrophoresis (SDS-PAGE) and then transferred to polyvinylidene difluoride (PVDF) membrane (Millipore, Bedford, MA, USA). After blocking with 5% Milk-TBS-Tween 20 at room temperature for 1 h, 1:800 diluted anti-VP2 monoclonal antibody (Ingenasa, Madrid, Spain) and 1:4000 diluted beta-actin monoclonal antibody (Thermo Fisher, Waltham, MA, USA) was added and then incubated overnight at 4 °C. The blots were further incubated with horseradish peroxidase (HRP)-conjugated goat anti-mouse IgG at 37 °C for 1 h. The SuperSignal™ West Pico PLUS Chemiluminescent Substrate Kit (Thermo Fisher, Waltham, MA, USA) was used for the detection of immunoreactive bands, and images were captured using an Amersham Imager 600 (GE Healthcare Life Sciences, Pittsburgh, PA, USA). Band intensities were measured with Image J software (Version 1.53), and the expression of the viral VP2 protein was compared with that of beta-actin.

### 2.5. Absolute Quantitative PCR

Absolute quantitative PCR was used to detect the copy number of the CPV in the above collected cell samples and to verify the inhibitory effect of NTZ on the different doses of the CPV and different times after CPV inoculation. Briefly, DNA was extracted using the Quick DNA Extraction Kit (Cwbio, Jiangsu, China). In the collected cells (approximately 4000 cells per sample), 40 µL of Solution A was added and vortexed for 20 s. After standing at room temperature for 3–5 min, 40 µL of Solution B was added, vortexed for further 30 s. The treated samples were diluted 400 times and stored at −20 °C for further use. Quantitative standard curves were prepared using laboratory-stored pMD-VP2S positive plasmid according to the previously reported method [13]. For sample quantification, 10 µL SuperReal PreMix Plus (SYBR Green) (TIANGEN, Beijing, China) and 0.2 µM specific primers (VP2-F 5′-CAAATAGAGCATTGGCTTAC-3′ and VP2-R 5′-TCCCATTTGAGTTACACCG-3′) were added to the 3 µL diluted sample, and the total volume was supplemented with water to 20 µL. Samples were amplified with the CFX Connect Real-Time PCR Detection System (Bio-Rad, Hercules, CA, USA). The samples were predenatured at 95 °C for 1 min, then denatured for 15 s at 95 °C and annealed/extended at 60 °C for 32 s. The denaturation, annealing and extension steps were cycled 40 times. The DNA copy number of the VP2-encoding gene in the sample was determined using the generated standard curve.

### 2.6. RNA Extraction and cDNA Library Construction and Sequencing

Two groups were set up in the experiment as follows: F81 cells were inoculated in a 6-well plate with 7.5 × 10^5^ cells/well. The drug-treated group treated cells with a 8 µM final concentration of NTZ, while the untreated control group treated cells with 0.1% of DMSO. After 1 h of treatment with NTZ or DMSO, the cells were infected with CPV SD6 (MOI of 0.5). Each group of samples had three biological replicates. After 40 h of virus infection, samples were collected and stored at −80 °C for future use. Transcriptome sequencing was conducted by LC Bio Technology CO., Ltd. (Hangzhou, China). Briefly, TRIzol (Thermo Fisher, Waltham, MA, USA) was used to separate and purify the total RNA of the sample according to the protocol provided by the manufacturer. Then, the amount and purity of total RNA were controlled using a NanoDrop ND-1000 (NanoDrop Technologies, Wilmington, DE, USA), and the integrity of the RNA was examined with a Bioanalyzer 2100 (Agilent, Santa Clara, CA, USA). The mRNA with PolyA (polyadenylate) was specifically captured using Dynabeads Oligo (dT) (Thermo Fisher, Waltham, MA, USA). The captured mRNA was fragmented and then synthesized into cDNA by SuperScriptTM II Reverse Transcriptase (Thermo Fisher, Waltham, MA, USA). The cDNA was further screened and purified, and the second-strand cDNA was further digested after end-repair, a base addition, and sequencing adapters ligation. The product was used as a template and amplified by PCR to construct a strand-specific library. The Illumina NovaseqTM 6000 (Illumina, San Diego, CA, USA) platform was then used for double-ended sequencing according to standard procedures.

### 2.7. Data Processing and Bioinformatics Analysis

The data generated by sequencing were preprocessed and filtered before being compared with the reference genome GCF_000181335.3_Felis_catus_9.0 (https://ftp.ncbi.nlm.nih.gov/genomes/all/GCF/000/181/335/GCF_000181335.3_Felis_catus_9.0 (accessed on 12 October 2023)), followed by a gene quantitative analysis. Genes or transcripts were quantified for each sample using StringTie (Version 2.1.6). Gene expression-level analysis mainly focuses on the protein-coding genes (mRNA) annotated by the genome. Gene expression levels were statistically analyzed using edgeR (Version 3.22.5) to evaluate the correlation of the gene expressions within and between groups, as well as the differentially expressed genes. Differential expression genes were screened in terms of differential multiple and significance level. In this study, genes with |log_2_FC| ≥ 1 and q < 0.05 in the NTZ-treated virus group compared to the 0.1%-DMSO-treated virus group were defined as differentially expressed genes. At the same time, a volcano map was drawn to analyze the overall distribution of differentially expressed genes. To further analyze the main biological functions of the DEGs, GO (http://geneontology.org (accessed on 18 October 2023)) functional enrichment was performed on the differentially expressed genes, and a GO enrichment classification histogram was drawn to reflect the distribution of the enriched terms in Biological Process (BP), Cellular Component (CC) and Molecular Function (MF). The KEGG database (http://www.genome.jp/kegg (accessed on 20 October 2023)) was used to analyze the signaling pathways enriched by the DEGs, and bubble plots were drawn based on the signaling pathway enrichment results. Volcano and heatmap, bar chart, and bubble plots were all drawn using GraphPad Prism (Version 9.0.0) software. The protein–protein interaction (PPI) network of identified differential genes were analyzed using a STRING online network (https://string-db.org/ (accessed on 24 October 2023)), and the results were visualized using Cytoscape software (Version 3.8.0).

### 2.8. Verifying the Expression Level of DEGs Using qRT-PCR

On the basis of the results of the GO, KEGG, and PPI analyses, some core genes related to cell cycle and division were selected from differential genes to verify the differential expression of DEGs. According to the method for preparing sequencing samples mentioned above, the drug-treated group and the control group without drug (with only 0.1% DMSO added) were collected. The collected samples were isolated and extracted RNA using RNeasy Mini kit (Qiagen, Hilden, Germany) according to the instructions. RNA was then reverse-transcribed into cDNA using the FastKing RT Kit (With gDNase) (TIANGEN, Beijing, China). The primers of these genes were designed based on the sequences obtained by Illumina sequencing, with β-actin as the internal reference gene, and the 2^−ΔΔC^_T_ method was used. The specific primers are shown in Table 1. Quantitative PCR was performed using SYBR Green. The amplification reaction solution contained 1 µL cDNA, 10 µL SuperReal PreMix Plus (SYBR Green) (TIANGEN, Beijing, China), 0.8 µL each of upstream and downstream primers, and the total volume was made up to 20 µL with water. The reaction solution was subjected to an initial denaturation for 1 min at 95 °C, followed by denaturation for 15 s at 95 °C, and then annealing/extension for 32 s at 60 °C, and cycle the denaturation, annealing, and extension steps 40 times. Amplification was performed using CFX Connect Real-Time PCR Detection System (Bio-Rad, Hercules, CA, USA).

### 2.9. Effect of Cell Cycle Checkpoint Kinase 1 (Chk1) Inhibitor on CPV Replication

F81 cells were inoculated into a 6-well plate with 7.5 × 10^5^ cells per well, and cells were pretreated with different concentrations of NTZ (S1627, 2.5 µM and 5 µM), MK-8776 (S2735, 2 µM and 4 µM) or Prexasertib HCl (S7178, 10 nM and 20 nM) for 1 h, respectively. While control cells were pretreated with 0.1% DMSO for 1 h. Cells were then inoculated with 0.1 MOI New CPV-2a strain SD6 (297Ala, 426Asn), and cell samples were collected 24 h later. The samples obtained were detected by Western blot according to the above method for VP2 expression. The CPV copy number in the sample was detected by absolute qRT-PCR to evaluate the inhibition of CPV by Chk1 inhibitors. In addition, the relative expression levels of genes CDC25B, CDC25C, CDC20, CCNA2 and CCNB1 related to the G2/M phase transition were detected by relative qRT-PCR. Primers are shown in Table 1.

### 2.10. Statistical Analysis

All statistical analysis experiments were set up with three replicates. Data were statistically analyzed using GraphPad Prism. The data analysis results are shown as the mean ± standard deviation (mean ± SD). Statistically significant differences were assessed using a Student’s *t*-test; * *p* < 0.05, ** *p* < 0.01, *** *p* < 0.005 and **** *p* < 0.001 were considered statistically significant.

## 3. Results

### 3.1. Effect of NTZ on Virus Replication

**Figure 1:**
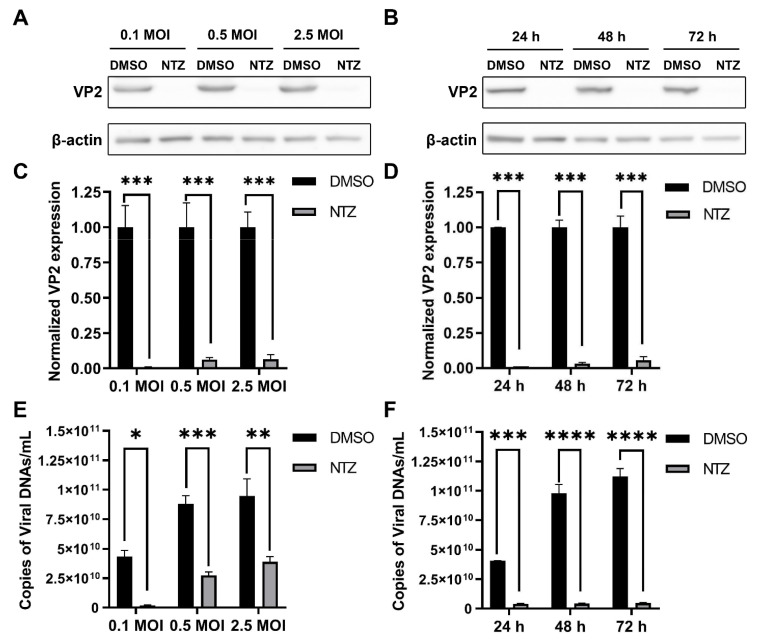
Inhibition of CPV replication by NTZ: (**A**,**C**) Western blot was used to detect the inhibitory effect of NTZ treatment on the infection of different doses of CPV (0.1 MOI, 0.5 MOI and 2.5 MOI) in F81 cells (**A**). The statistical graph obtained from the calculation of the intensity bands in experiment A (**C**); (**B**,**D**) Western blot was used to detect the inhibitory effect of NTZ treatment on the infection of CPV in F81 cells at 24 h, 48 h and 72 h postinfection (**B**). The statistical graph obtained from the calculation of the intensity bands in experiment B (**D**). (**E**) Quantitative real-time PCR assay was used to detect the inhibitory effect of NTZ treatment on the infection of different doses of CPV (0.1 MOI, 0.5 MOI and 2.5 MOI) in F81 cells, and the number of virus copies per milliliter on CPV-infected F81 cells were calculated. (**F**) Quantitative real-time PCR assay was used to detect the inhibitory effect of NTZ treatment on the infection of CPV in F81 cells at 24 h, 48 h and 72 h postinfection, and the number of virus copies per milliliter on CPV-infected F81 cells were calculated; * *p* < 0.05, ** *p* < 0.01, *** *p* < 0.005 and **** *p* < 0.001 were considered statistically significant.

In a previous study, we reported that NTZ can inhibit the replication of different subtypes of CPV strains in a dose-dependent manner. In this study, after NTZ drug treatment, we infected F81 cells with different inoculation doses (inoculation doses of 0.1, 0.5 and 2.5 MOI). Western blot analysis revealed that the expression of VP2 decreased to 0.009 ± 0.003%, 0.062 ± 0.024%, and 0.064 ± 0.055%, respectively, compared with the 0.1%-DMSO-treated virus group (Figure 1A,C). qRT-PCR detection found that under different doses of inoculation the copy number also significantly reduced from 4.34 ± 0.93 × 10^10^, 8.79 ± 1.21 × 10^10^ and 9.47 ± 2.53 × 10^10^ copies/mL to 0.18 ± 0.08 × 10^10^, 2.75 ± 0.48 × 10^10^ and 3.9 ± 0.74 × 10^10^ copies/mL (Figure 1E), respectively. The results show that NTZ exhibited very good inhibitory effects on different doses of CPV infection. Even when the inoculation dose was 2.5 MOI, NTZ was able to inhibit the virus effectively. In addition, we found that after NTZ drug treatment and virus inoculation, NTZ still had a good inhibitory effect on CPV at 24 h, 48 h and 72 h after virus infection. Western blot analysis results of samples at different time points revealed that the expression of VP2 decreased to 0.012 ± 0.0004%, 0.031 ± 0.015% and 0.056 ± 0.046%, respectively, compared with the 0.1%-DMSO-treated virus group (Figure 1B,D). Similarly, qRT-PCR detection of samples at different time points showed that the copy number also significantly reduced from 4.05 ± 0.03 × 10^10^, 9.77 ± 1.32 × 10^10^ and 11.21 ± 1.19 × 10^10^ copies/mL to 0.4 ± 0.07 × 10^10^, 0.42 ± 0.08 × 10^10^ and 0.47 ± 0.05 × 10^10^ copies/mL (Figure 1F), respectively. Even 72 h postinfection, the inhibitory effect was very significant. All of the data indicate that NTZ is an effective small-molecule drug against CPV.

### 3.2. Overview of Transcriptome Sequencing Data

The prepared two groups of samples were sequenced using the Illumina NovaseqTM 6000 platform, and the sequencing data were further analyzed for gene expression levels after quality control. The Q30 percentage of clean data was greater than 97.52% for all samples, and the GC content of clean data ranged from 45% to 50% for all samples (Table 2). The 0.1%-DMSO-treated virus group obtained more than 37,459,332 clean reads. At least 51.18% of the clean reads can be mapped to the genome, and at least 49.18% of the clean reads can be uniquely mapped to the genome. The NTZ-treated virus group obtained more than 37,896,286 clean reads. At least 87.4% of the clean reads were successfully mapped to the reference genome (Table 2). After splicing and merging the transcripts of each sample using StringTie (2.1.6), 17,281 genes were identified in the 0.1%-DMSO-treated virus group, and 17,873 genes were identified in the NTZ-treated virus group.

### 3.3. Analysis of Differentially Expressed Genes

**Figure 2:**
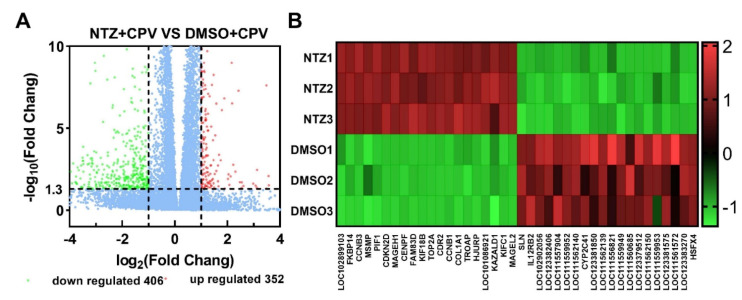
Changes in differentially expressed genes (DEGs) between F81 cells treated with 0.1% DMSO and NTZ (CPV was inoculated 1 h after treatment). (**A**) Differentially expressed genes (DEGs) screened with |log_2_FC| ≥ 1 and q < 0.05. Red, green and blue dots represent upregulated, downregulated and no significant differential genes, respectively. (**B**) Heat map analysis of the top 40 differentially expressed genes. Red indicates upregulated DEGs, while blue indicates downregulated DEGs. RNA-seq was performed on samples from three independent replicate experiments.

In order to study the changes in the expression profile of the sequenced samples, the FPKM expression values of each sample’s genes or transcripts were measured using StringTie software, Version. 2.2.1. DESeq2 software was used to calculate the FC (fold change) values of each gene between the 0.1%-DMSO-treated virus group and the NTZ-treated virus group, and genes with a more than two-fold change were defined as DEGs (|log_2_FC| ≥ 1 and q < 0.05). Compared with the virus-infected control group (cells pretreated with 0.1% DMSO group), the virus-infected cells pretreated with the NTZ group produced 352 upregulated genes and 406 downregulated genes (volcanic map) (Figure 2A). In the virus-infected cells pretreated with the NTZ group, several genes encoding products involved in the host cell cycle were upregulated, including CCNA2, CCNB1, CCNB2 and CCNB3, as well as the cell division cycle proteins CDC20, CDC25B, CDC25C, CDCA3, CDCA2 and CDCA8. In addition to the above genes, several other genes related to mitosis and cell cycle processes were also upregulated, including CDKN3, FOXM1, CENPI, MYC and DBF4GTSE1 (Figure 2 and Supplementary Table S1).

### 3.4. Enrichment Analysis of GO Terms and KEGG Pathways

**Figure 3:**
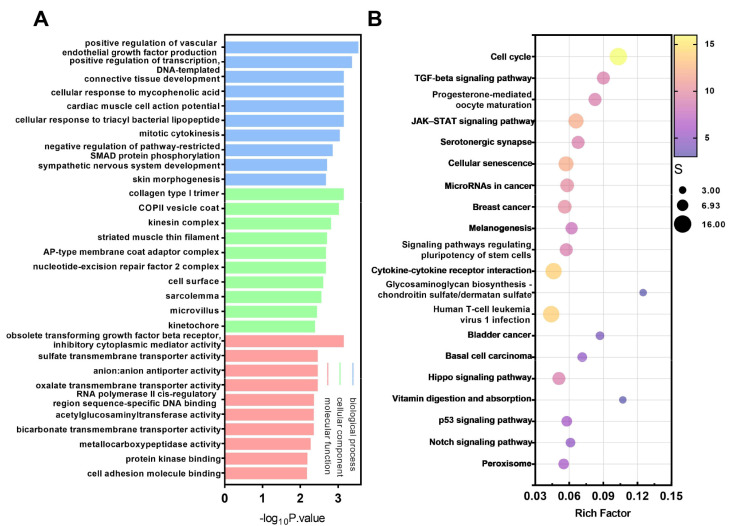
GO and KEGG enrichment analyses of differentially expressed genes (DEGs) between F81 cells treated with 0.1% DMSO and NTZ (CPV was inoculated 1 h after treatment). (**A**) The top 30 Gene Ontology (GO) terms of differentially expressed genes. The GO term is located on the x-axis. The enrichment rate of the GO terms related to BP, CC and MF is displayed. (**B**) The top 20 pathways from the Kyoto Encyclopedia of Genes and Genomes (KEGG) enrichment analysis of the differentially expressed genes. The name of the KEGG pathway is on the x-axis. The size of bubbles is the gene enrichment rate.

In order to identify the function of differentially expressed genes, we performed GO enrichment analysis on differentially expressed genes in the 0.1%-DMSO-treated virus group and the NTZ-treated virus group. The GO enrichment analysis was divided into three categories: Biological Process (BP), Cellular Component (CC), and Molecular Function (MF). The top 30 GO entries are shown in Figure 3A. The enrichment results show that the differentially expressed genes identified are involved in a variety of biological processes, including positive regulation of vascular endothelial growth factor production, positive regulation of transcription, connective tissue development, mitotic cytoplasmic division and negative regulation of pathway restricted SMAD protein phosphorylation. In addition, the increase in vascular endothelial growth factor can promote cell proliferation and differentiation [22]. In summary, the GO annotations indicate that inhibition of CPV replication by NTZ drug treatment may involve interactions between processes such as mitosis, cell proliferation and differentiation. To further identify the pathways related to signal transduction and biochemical metabolism of differentially expressed genes, we also conducted KEGG pathway enrichment analysis. These DEGs involved a total of 257 pathways, and Figure 3B shows the 20 most representative enriched pathways. It is worth noting that DEGs are significantly enriched in the cell cycle, TGF-β signaling pathway and JAK-STAT signaling pathway, indicating that the identified differentially expressed genes play a crucial role in cell growth, proliferation and division.

### 3.5. Protein–Protein Interaction (PPI) Network Analysis of DEGs

**Figure 4:**
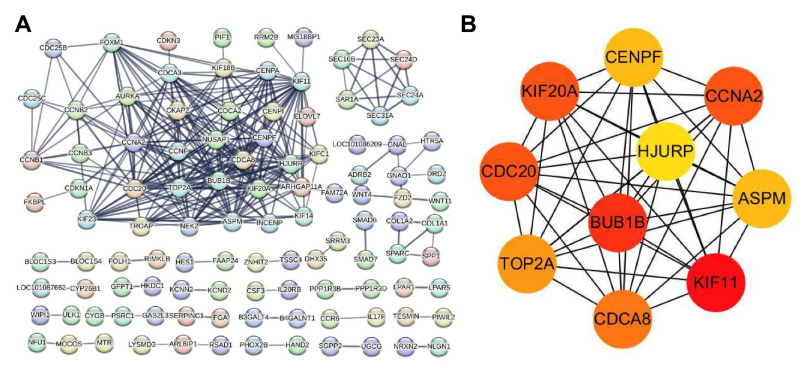
Protein–protein interaction network analysis of the differentially expressed genes (DEGs) between the F81 cells treated with 0.1% DMSO and NTZ (CPV was inoculated 1 h after treatment). (**A**) The STRING database (http://string-db.org/, accessed on 21 November 2023) was used to analyze the protein–protein interaction networks based on the proteins corresponding to the identified DEGs. The protein interaction relationships of the identified DEGs present in the database were extracted for the network’s construction. (**B**) The top 10 genes were screened out for protein interactions using the Cytoscape (v3.10.0) degree algorithm.

In order to further understand the interactions among the DEG-encoded proteins, a PPI network analysis of the DEGs was performed using the STRING database. The PPI network map was used to identify the central genes involved in the antiviral process in the DEGs (Figure 4A). The analysis results show that the genes related to the cell cycle and division are closely related, with BUB1B having the highest centrality, followed by KIF20A, KIF11, CDCA8, TOP2A, ASPM, CCNA2 and CDC20 (Figure 4B). The constructed protein interaction relationships indicate that in the NTZ-treated virus group, drug treatment is involved in the changes in the cell cycle and cell division process of the host cells.

### 3.6. Differential Expression of Enriched or Associated Genes

**Figure 5:**
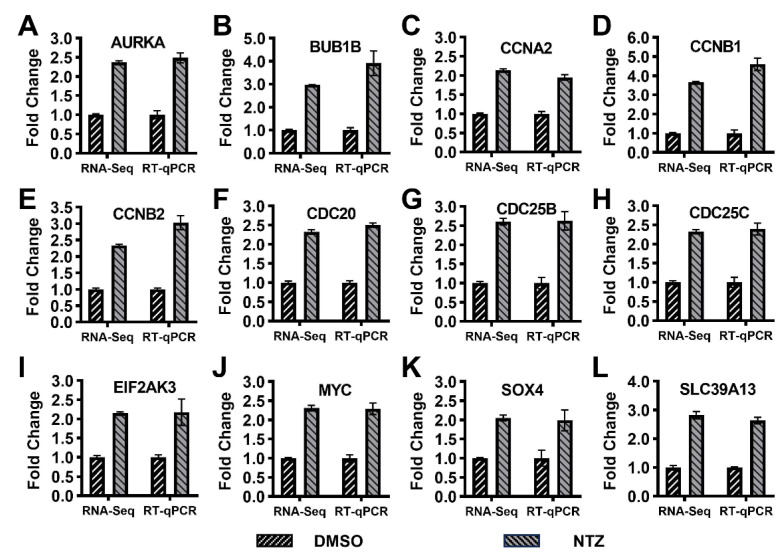
Validation of the expression of differentially expressed genes (DEGs) identified using RNA-seq sequencing: (**A**–**L**) qRT-PCR versus RNA-seq analyses of the selected twelve genes (AURKA, BUBIB, CCNA2, CCNB1, CCNB2, CDC20, CDC25B, CDC25C, EIF2AK3, MYC, SOX4 and SLC39A13). The β-actin gene was used as an internal control. The relative fold changes in each gene expression were calculated using the comparative 2^−ΔΔC^_T_ method.

Among the DEGs obtained from transcriptome sequencing, some genes enriched in or associated with the GO, KEGG, and PPI analyses were selected. The selected 12 genes were associated with cell cycle, cell division and cell proliferation and differentiation. We examined the differential expression levels of related genes in the 0.1%-DMSO-treated virus group and the NTZ-treated virus group. As shown in Figure 5, the results of both the qRT-PCR and RNA-seq showed that all of these genes are upregulated to varying degrees, and of the 12 genes tested, the differential changes obtained using RNA-seq and qRT-PCR were AURKA (2.369 vs. 2.485), BUBIB (2.972 vs. 3.911), CCNA2 (2.138 vs. 1.949), CCNB1 (3.666 vs. 4.606), CCNB2 (2.332 vs. 3.028), CDC20 (2.324 vs. 2.504), CDC25B (2.604 vs. 2.627), CDC25C (2.324 vs. 2.391), EIF2AK3 (2.153 vs. 2.175), MYC (2.31 vs. 2.291), SOX4 (2.048 vs. 1.990) and SLC39A13 (2.825 vs. 2.637), respectively, and the trend in the results is consistent.

### 3.7. Cell Cycle Checkpoint Kinase 1 (Chk1) Inhibitors Inhibit CPV Replication

**Figure 6:**
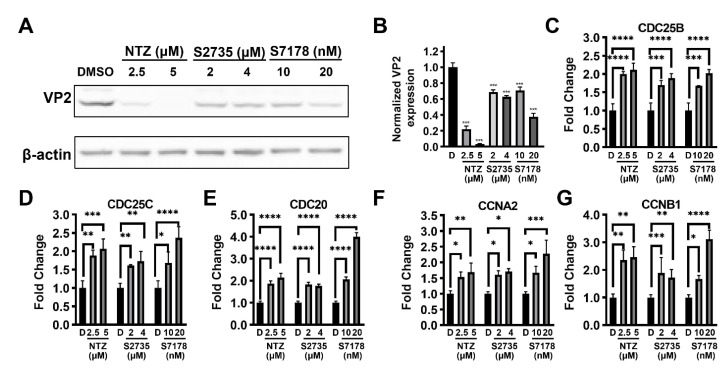
Inhibition of CPV in F81 cells by cell cycle checkpoint kinase 1 (Chk1) inhibitors and their effects on the expression of G2/M phase transition-related genes. (**A**) Western blot was used to detect the effects of the NTZ (S1627, 2.5 µM and 5 µM), S2735 (MK-8776, 2 µM and 4 µM) and S7178 (Prexasertib HCl, 10 nM and 20 nM) treatments on CPV replication. “DMSO” represents the control group treated with 0.1% DMSO. (**B**) The statistical graph obtained from the calculation of the intensity bands in experiment A. “D” represents the control group treated with 0.1% DMSO. (**C**–**G**) qRT-PCR was used to detect the effects of the NTZ (S1627, 2.5 µM and 5 µM), S2735 (MK-8776, 2 µM and 4 µM) and S7178 (Prexasertib HCl, 10 nM and 20 nM) treatments on the expression of the CDC25B (**C**), CDC25C (**D**), CDC20 (**E**), CCNA2 (**F**) and CCNB1 (**G**) genes associated with G2/M phase transition. “D” represents the control group treated with 0.1% DMSO. The β-actin gene was used as an internal control. The relative fold changes in each gene expression were calculated using the comparative 2^−ΔΔC^_T_ method; * *p* < 0.05, ** *p* < 0.01, *** *p* < 0.005 and **** *p* < 0.001 were considered statistically significant.

From the analyses of the differential genes using GO, KEGG, and PPIs mentioned above, it was found that in the process of the NTZ-mediated anti-CPV effect, there were obvious changes in the genes or pathways related to the cell cycle, mitosis and cell proliferation and differentiation. It was found that when cells were treated with the cell cycle checkpoint kinase 1 (Chk1) inhibitor MK-8776 (S2735) or Prexasertib HCl (S7178), they all showed certain anti-CPV ability. Compared with the 0.1% DMSO control group, treatment with 2.5 µM and 5 µM of NTZ reduced the expression of VP2 to 22.17 ± 7.07% and 3.26 ± 1.58%, respectively, while treatment with 2 µM and 4 µM of MK-8776 reduced the expression of VP2 to 68.5 ± 5.35% and 62.86 ± 2.49%, respectively. Treatment with 10 nM and 20 nM of Prexasertib HCl reduced the expression of VP2 to 70.8 ± 7.52% and 37.65 ± 7.58%, respectively (Figure 6A,B). In addition, the detection of G2/M phase transition-related genes in the two inhibitor-treatment groups showed that the relative expression levels of CDC25B, CDC25C, CDC20, CCNA2 and CCNB1 increased to varying degrees compared with that of the 0.1% DMSO control group, which is in line with the trend of the NTZ treatment group, as expected (Figure 6C–G).

## 4. Discussion

Among canine diseases, viral diarrhea causes serious harm to the canine industry and canines due to its complex etiology and high morbidity. CPV is an important cause of death and morbidity in dogs with viral diarrhea, especially puppies [9,23]. Mutation, recombination and co-infection have been shown to exacerbate clinical symptoms and pose challenges to the prevention and control of CPV infection [24]. Therefore, an in-depth understanding of the molecular mechanisms of antiviral drugs is of great significance for inhibiting the occurrence and prevalence of CPV infection. Previously, we screened the anti-CPV drug NTZ from the FDA-approved drug library based on the CPE-based trial. It was found that the 50% antiviral efficacy concentration (EC50) of NTZ against CPV was 2.71 µM, and its 50% cytotoxicity concentration (CC50) was 21.02 µM. In addition, when cells were pretreated with 10 µM NTZ for 1 h and then inoculated with CPV, the virus inhibition efficiency was 98.09 ± 9.97% after 40 h of virus inoculation, and the corresponding cell viability analysis of NTZ-treated but uninfected cells showed that approximately 95.23 ± 0.53% of the cells survived, indicating no obvious toxicity was observed at this concentration [13]. Therefore, it is critical to explore the antiviral mechanism of NTZ to provide new therapeutic targets or strategies for CPV [13].

Our previous study found that NTZ inhibited the replication of CPV. According to a report by Pollock, R. V. and M. J. Coyne, the minimum infection dose of CPV may be as low as 100 50% tissue culture infective doses (TCID_50_) [25], while the 0.1 MOI, 0.5 MOI and 2.5 MOI doses used in this study are equivalent to inoculating each well of the 6-well plate with 10^5^ TCID_50_, 5 × 10^5^ TCID_50_ and 2.5 × 10^6^ TCID_50_, respectively. NTZ inhibited the virus well at an inoculation dose of 2.5 MOI. In addition, even 72 h postinfection, NTZ still had a good inhibitory effect on CPV. Therefore, NTZ demonstrated an excellent anti-CPV effect. It is worth noting that NTZ has been investigated as a therapeutic option for feline calicivirus virulent systemic disease (FCV-VSD) [26]; therefore, the development of NTZ-based treatment for CPV infection holds great prospect.

The analysis of differential transcription data between CPV-infected and -negative uninfected groups found that CPV infection activates the expression of membrane-associated genes related to antigen processing and presentation pathways, thereby activating immunity. However, pathways related to amino acid synthesis and metabolism were inhibited [27,28]. Moreover, studies have shown that NTZ has broad-spectrum antiviral activity. NTZ can inhibit the replication of Flaviviridae viruses, such as dengue fever virus, yellow fever virus and Japanese encephalitis virus, by inhibiting viral adsorption and entry [16]. NTZ is able to induce cholesterol-25-hydroxylase (CH25H), which reduces intracellular cholesterol levels and directly affects viral replication [29]. In addition, NTZ treatment can counteract the blocking effect of EBOV VP35 protein on the recognition of EBOV by RIG-I and PKR, enhance innate immune response and thereby inhibit the replication of EBOV in human cells [30]. Research on anti-influenza viruses has shown that NTZ inhibits the replication of 16 strains of H1N1 influenza and 9 strains of influenza B. The anti-influenza mechanism of NTZ is by the inhibition of the maturation of viral hemagglutinin in the post-translational step [16]. NTZ also impedes terminal glycosylation of SARS-CoV-2 spike protein and reduces the infectivity of progeny virus particles and cell fusion [31]. In summary, NTZ can mediate its antiviral activity by modulating host innate immunity or by directly targeting viral targets [32]. The relevant research provides us with a good reference.

The molecular mechanism of NTZ anti-CPV is still unclear. In order to elucidate the molecular mechanism by which NTZ inhibits CPV replication in vitro, 758 DEGs were identified in this study by the high-throughput sequencing of a 0.1%-DMSO-treated virus group and a NTZ-treated virus group. The GO and KEGG enrichment analyses of the differential genes revealed the enrichment of pathways related to cell cycle, mitosis, cell growth differentiation and apoptosis, including the TGF-β signaling pathway, JAK-STAT signaling pathway, Hippo signaling pathway, p53 signaling pathway and Notch signaling pathway. Previous studies have reported that CPV can induce cell cycle arrest [33,34,35,36]. The cellular DNA damage response triggered by the invading single-stranded parvovirus genome can induce cell cycle arrest in the S or G1 phase in parvovirus-infected cells. The prolonged S phase provides the virus with sufficient DNA and protein resources for rapid replication [37]. The analysis of the high-throughput results in this study found that NTZ can alter the cell cycle and further inhibit viral reproduction [38].

Further research found that when the F81 cells were treated with cell cycle checkpoint kinase 1 (Chk1) inhibitors MK-8776 (S2735) or Prexasertib HCl (S7178), both inhibitors inhibited CPV. And both inhibitor treatment groups, like the NTZ treatment group, were able to upregulate the expression of G2/M phase transition-related genes compared to the 0.1% DMSO control group. On the one hand, this indicates that the regulation of the cell cycle, especially G2/M phase transition, plays an important role in the NTZ anti-CPV process. On the other hand, the anti-CPV effect of the two inhibitors was still lower than that of NTZ. This indicates that there are still other mechanisms mediating the inhibition of CPV by NTZ. For example, NTZ may directly affect the transcription and expression levels of the virus itself. In addition, further research is needed on the direct or indirect pathway through which NTZ regulates the cell cycle and cell division, thereby inhibiting CPV replication.

## 5. Conclusions

In conclusion, in this study, we found that NTZ pretreatment significantly inhibited CPV replication in F81 cells. Through high-throughput RNA sequencing analysis, multiple differentially expressed genes related to the cell cycle and mitosis were identified. Further GO and KEGG enrichment analyses also indicated that differential genes were enriched in the cell cycle, mitosis, cell growth differentiation and other related pathways. A protein–protein interaction (PPI) network analysis also identified multiple hub genes related to the cell cycle and mitosis. The expression levels of related differential genes were verified by qRT-PCR and were consistent with the sequencing results. This study also shows that the regulation of the cell cycle plays an important role in the inhibition of the CPV. Our findings provide new insight into the prevention and control of the CPV.

## Figures and Tables

**Table 1:** Primers used in this study.

Gene ID	Primers	Sequence 5′-3′	Size (bp)
101091184	AURKA-F	TCGTCTCCAGCCATAAACCG	86
	AURKA-R	CAGCGGCCTAGAGACAGAAC	
101087670	BUB1B-F	AAATGATCCTCTGGATGTTTGG	184
	BUB1B-R	GCATAAATGCCCCAATTTGAGCC	
101088573	CCNA2-F	GGATGGTAGTTTTGAGTCACCAC	202
	CCNA2-R	CACAAGGATAGCCCTCATACTGT	
101086284	CCNB1-F	TCGGAGACATTGGTAACAAAGTC	208
	CCNB1-R	ATAGGCTCCGGAGAAAGCTTTT	
101080972	CCNB2-F	CCGACGGTGTCCACTGATTT	180
	CCNB2-R	ATTTGTTTTGGCGGGTTGAACA	
101097259	CDC20-F	GCTTTGAATCTGAACGGCTTTG	77
	CDC20-R	TCTGGGGCATTTTGTGGTTTT	
101092757	CDC25B-F	GGCCGAGGAACCTAAAGCCCG	139
	CDC25B-R	CTTCCCATCCACAGTCTGCAGAA	
101086942	CDC25C-F	CCGCTATCCATATGAGTACCAG	147
	CDC25C-R	AATTCACAGTGGAACACGA	
101095973	EIF2AK3-F	GGAAACTAGAGCCGGATTTATT	111
	EIF2AK3-R	ACTGTGTCCATCATGGCAGCTTC	
100379628	MYC-F	CCCCTCCACTAGGAAGGAC	96
	MYC-R	CTGATGCATTTGCGGTTGTTG	
111560412	SOX4-F	GACCTGCTCGACCTGAACC	107
	SOX4-R	CCGGGCTCGAAGTTAAAATCC	
101095193	SLC39A13-F	TCAGTGGCTATCTCAACCT	100
	SLC39A13-R	AGGAGCCCAATCTTCTTGCT	
101098507	ACTB-F	CATGTACGTGGCCATCCAGGC	250
	ACTB-R	CTCCTTGATGTCACGCACAAT	

**Table 2:** Summary of the quality control of the sample sequencing data.

Sample	Raw Reads	Clean Reads	Valid Ratio (%)	Q30 (%)	GC Content (%)	Mapped Reads	Unique Mapped Reads
DMSO 1	38,970,132	37,459,332	96.12	97.77	45	19,169,896 (51.18%)	18,422,453 (49.18%)
DMSO 2	39,582,814	38,127,200	96.32	97.52	45.50	20,344,108 (53.36%)	19,557,030 (51.29%)
DMSO 3	39,418,474	38,071,304	96.58	97.58	45	20,064,280 (52.70%)	19,315,191 (50.73%)
NTZ 1	39,292,554	37,896,286	96.45	97.83	50	33,196,056 (87.60%)	31,342,275 (82.71%)
NTZ 2	39,735,696	38,298,006	96.38	98.00	49.50	33,471,408 (87.40%)	31,946,335 (83.42%)
NTZ 3	41,529,962	40,036,868	96.40	97.91	50	35,142,284 (87.77%)	33,508,501 (83.69%)

## Data Availability

Data are contained within the article and supplementary materials.

## Supplementary Materials

The following supporting information can be downloaded at: https://www.mdpi.com/article/10.3390/v16020282/s1, Table S1: 758 differentially expressed genes between the 0.1%-DMSO-treated virus group and the NTZ-treated virus group.
